# Yield Response of Spring Maize to Inter-Row Subsoiling and Soil Water Deficit in Northern China

**DOI:** 10.1371/journal.pone.0153809

**Published:** 2016-04-21

**Authors:** Zhandong Liu, Anzhen Qin, Ben Zhao, Syed Tahir Ata-Ul-Karim, Junfu Xiao, Jingsheng Sun, Dongfeng Ning, Zugui Liu, Jiqin Nan, Aiwang Duan

**Affiliations:** 1 Key Laboratory of Crop Water Use and Regulation, Ministry of Agriculture, Farmland Irrigation Research Institute, Chinese Academy of Agricultural Sciences, Xinxiang, China; 2 National Engineering and Technology Center for Information Agriculture, Jiangsu Key Laboratory for Information Agriculture, Jiangsu Collaborative Innovation Center for Modern Crop Production, Nanjing Agricultural University, Nanjing, China; University of Vigo, SPAIN

## Abstract

**Background:**

Long-term tillage has been shown to induce water stress episode during crop growth period due to low water retention capacity. It is unclear whether integrated water conservation tillage systems, such asspringdeepinter-row subsoiling with annual or biennial repetitions, can be developed to alleviate this issue while improve crop productivity.

**Methods:**

Experimentswere carried out in a spring maize cropping system on Calcaric-fluvicCambisolsatJiaozuoexperimentstation, northern China, in 2009 to 2014. Effects of threesubsoiling depths (i.e., 30 cm, 40 cm, and 50 cm) in combination with annual and biennial repetitionswasdetermined in two single-years (i.e., 2012 and 2014)againstthe conventional tillage. The objectives were to investigateyield response to subsoiling depths and soil water deficit(SWD), and to identify the most effective subsoiling treatment using a systematic assessment.

**Results:**

Annualsubsoiling to 50 cm (AS-50) increased soil water storage (SWS, mm) by an average of8% in 0–20 cm soil depth, 19% in 20–80 cm depth, and 10% in 80–120 cm depth, followed by AS-40 and BS-50, whereas AS-30 and BS-30 showed much less effects in increasing SWS across the 0–120 cm soil profile, compared to the CK. AS-50 significantly reduced soil water deficit (SWD, mm) by an average of123% during sowing to jointing, 318% during jointing to filling, and 221% during filling to maturity, compared to the CK, followed by AS-40 and BS-50. An integrated effect on increasing SWS and reducing SWD helped AS-50 boost grain yield by an average of 31% and biomass yield by 30%, compared to the CK. A power function for subsoiling depth and a negative linear function for SWD were used to fit the measured yields, showing the deepest subsoiling depth (50 cm) with the lowest SWD contributed to the highest yield. Systematic assessment showed that AS-50 received the highest evaluation index (0.69 out of 1.0) among all treatments.

**Conclusion:**

Deepinter-row subsoilingwith annual repetition significantly boosts yield by alleviating SWD in critical growth period and increasing SWS in 20–80 cm soil depth. The results allow us to conclude that AS-50 can be adopted as an effective approach to increase crop productivity, alleviate water stress, and improve soil water availability for spring maize in northern China.

## Introduction

Food security has been a global issue due to the ever-growing population, and largely dependson substantial yield increase to meet the food requirement [1]. Maize(*Zea mays* L.), the second largest crop on the planet, contributes 30% nation’scereal production, and 74% livestock consumption [2,3]. Of this, about 56% maize planting area was cultivated withrainfed farming [4]. In arid and semi-arid areas of northern China, low and variable rainfall and improper tillage management often causewater stress in crop growth period, andresult in yield fluctuations from year to year [5]. Long-term conventional tillagegenerally increases risk of plow pan in subsoils, which in turn hastens water stress [6]. In the past decade, occurrence of water stress became frequent and the severity intensified due to the global climate change. The resultant decline in crop productivity has raised serious concerns and emphasized the need to develop soil tillagestrategiesto improve crops adaptability to water stress [7]. Conservation tillage, including subsoiling, is widely adopted to retain soil moistureby improving soil physical properties (e.g., soil aeration, soil porosity, and soil permeability)[8–10]. In particular, subsoiling is the most effective strategy used to break plow pan, and is generally adopted as a solution to potential soil compaction induced by long-term conventional tillage or no-tillage. Moreover, italso increases water availability by facilitating root exploration in soils prone to compaction [11].

Conventional tillage is usually performed after harvest for the next year’s seed bed preparation using harrowing plus moldboard plowing with the removal of crop residues. It is not only economic expensive, but also risks soil health [12]. Previous studies reported that long-term conventional tillage markedly reduced soil porosity, water holding capacity, and rainwater infiltration in topsoil (0–30 cm) in sandy loam soils [11], silt loam soils [13], and silty clay soils [14]. The detrimental effect finally hindered water and nutrient uptake and retarded plant growth [15].

Compared to conventional tillage, subsoiling has less soil disturbance due to reduced vehicle traffic frequency. It alsopermits large root penetration by breaking the plow pan [16]. Although energy cost increases withsubsoilingdepthincreasing, deep subsoiling has been proven to improvelodging resistance of spring maize, and isimplemented as an alternative to cope with increasing events of natural hazards(i.e., water-logging, storm, and drought, etc.) in northern China [17]. Moreover, it also offers a conciliation between conventional and zero tillage by loosening subsoilsand permitting covers of crop residues [18,19]. During the critical growth period, subsoilingevery year can alleviatewater stress viainfiltrating more rainwater and retaining more soil moisture in soil layers compared to conventionaltillage [20]. However, subsoilingin alternate yearsshows non-significant effect on the soil water status and grain yield as compared totheconventional tillage [9].

Currently, researchers have achieved significant information regarding the effects of subsoiling on soil physical properties, soil moisture, root morphology and grain yields [9,11,21]. However, the information regarding the integratedeffects of subsoiling depth and repetition on water- and yield-related factors of spring maizeis still lacking. Moreover, best to our knowledge, no attempt was made to quantify the yield response to subsoiling depth, nor the relationship between yield and soil water deficit (SWD). In this study, we hypothesized that annual and biennial subsoiling coupled with different subsoilingdepthssignificantly influenced grain and biomassyielddue to their joint manipulating effects on SWDand soil water storage (SWS) and that the yield changed with subsoiling depths.

The objectives of this study were to (i) investigate the effects of different subsoiling depths and repetitionsonSWS, SWD and yield, (ii) quantify the relationship between yield and subsoiling depth, and between yield and SWD, and (iii) identify the most effectivesubsoilingsystem in spring maize of northern China using an evaluation index.

## Materials and Methods

### Site description

**Fig 1:**
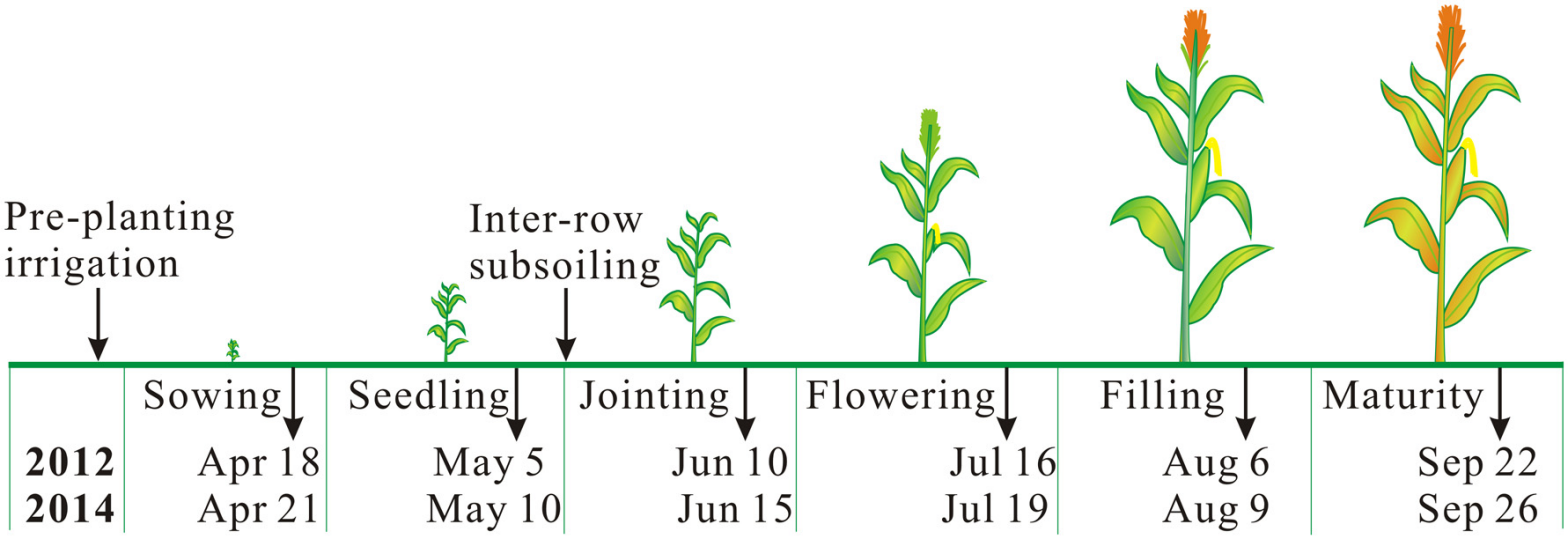
Seasonal schedule of crop growth stages, pre-planting irrigation and inter-row subsoiling for spring maize in the field experiment. The date in the figure was recorded when more than three quarters (>75%) of the crops developed into the particular growth stage.

**Fig 2:**
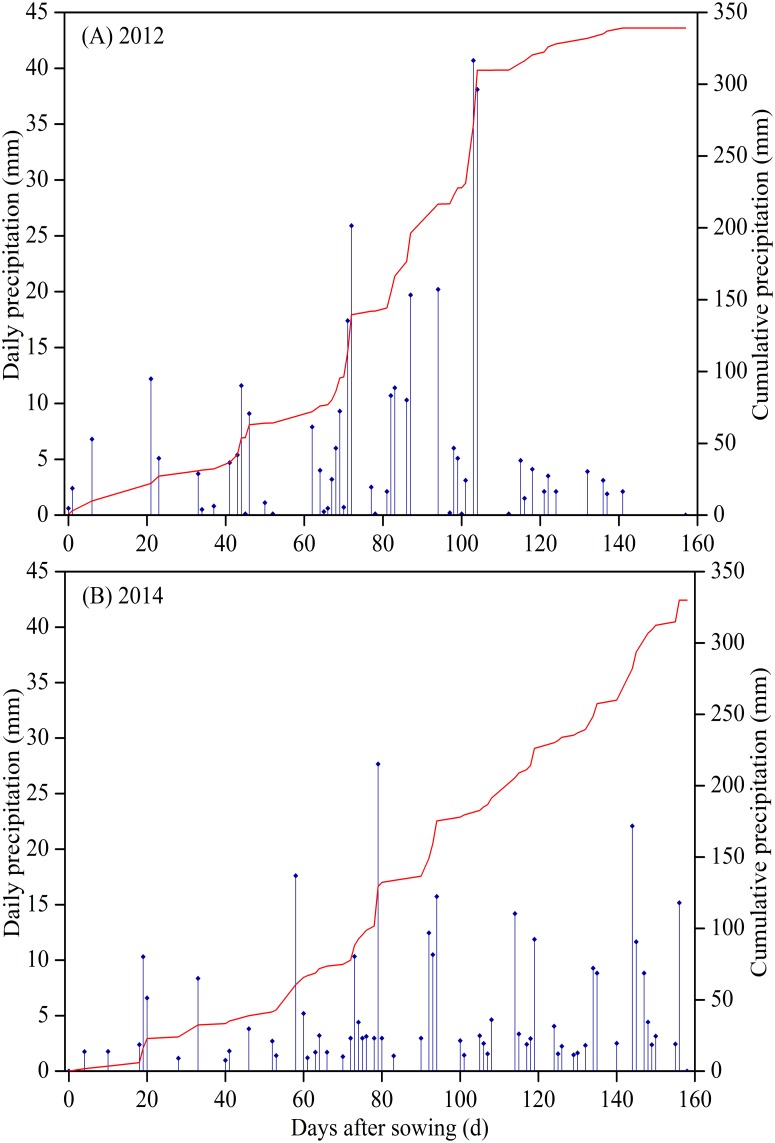
Daily and cumulative precipitation (mm) from April to September in (A) 2012 and (B) 2014, at Jiaozuo Experiment Station, China. Vertical drop lines represent daily precipitation, and curve lines stand for cumulative precipitation.

The field experiment was carried out at Jiaozuo experiment station (112°55′ E, 35°40′ N; 150 m a.s.l.) from 2009 to 2014. The station is affiliated to Farmland Irrigation Research Institute, Chinese Academy of Agricultural Sciences, in Henan Province, northern China. Permissions were granted to the authors from the institute to conduct the experiment at the station. Long-term (1981–2010) mean annual precipitation, temperature, frost-free period, and sunshine hours were494 mm, 10.5°C, and206 d, 2300 h, respectively. The soil is classified as Calcaric-fluvicCambisols [22]. Soil fertility (i.e., N, P_2_O_5_, SOC)in 0–40 cm soil layer, and soil physical properties(Table 1) in 0–120 cm soil layer were determined at the beginning of the experiment (mid April, 2009). Total nitrogen (N) was1.26 g kg^-1^, measuredusing Kjeldahldetermination. Alkali-hydrolyzablenitrogen was101.5 mg kg^-1^, measured using alkali-hydrolyzableproliferation method. Available phosphorus was41.2 mg kg^-1^, measuredusing sodium bicarbonate method. Soil organic carbonwas9.3 g kg^-1^, determined followingdichromate oxidation procedure. The field was previously managed using notillagein 2003 to 2008. Seasonal schedule of crop growth stage, pre-planting irrigation and inter-row subsoiling in the experiment were presented in Fig 1. Daily and cumulativeprecipitation monitored by a local weather station was presented in Fig 2. Soil bulk density, determined using stainless steel ringmethod, wilting point and field capacity (the determination method was described in measurement and calculation section), and soil particle-size analysis based on the USDA textural soil classification system [22]were presented in Table 1.

**Table 1:** Physical properties and taxonomic classification of the studied soils prior to the start of the experiment, at Jiaozuo experiment station, northern China.

Depths	Soil bulk density	Wilting point	Field capacity	Sand (>0.05 mm) ^a^	Silt (0.002–0.05 mm)	Clay (<0.002 mm)	Soil texture ^b^
	– g cm^-3^ –	– – – cm^3^ cm^-3^ – – –		– – – – – – – – – – g kg^-1^ – – – – – – – – – –			
**0–20**	1.35	13.3	31.5	694	279	27	Sandy loam
**20–40**	1.41	13.7	31.9	712	265	23	Sandy loam
**40–60**	1.48	14.5	32.7	726	238	36	Sandy loam
**60–80**	1.43	15.6	33.6	828	161	11	Loamy sand
**80–100**	1.46	14.4	34.2	852	105	43	Loamy sand
**100–120**	1.4	13.4	29.8	933	54	13	Sand

^a^ Soil particle fraction based on the USDA textural soil classification system.

^b^ Soil texture is determined using the soil particle percentage.

### Experimental design

The experiment was conducted by arranging the treatments in a randomized split blocks design having 3 replicates. The size of each plot was 64 m^2^ (8m × 8 m). Subsoiling depths (30 cm, 40 cm, and 50 cm) were arranged in main plots while the annual subsoiling (AS) and biennial subsoiling (BS) were arranged in subplots. Conventional tillage was performed to a depth of 20 cm using a moldboard plow, followed by harrowing, and was used as control (CK). The size of the buffer area, used as an aisle between subplots, was 1 m wide and 24 m long. There was no buffer area between the main plots. The tillage treatments showed less treatment effect in first few years of experiment, therefore, the selective data obtained from the fourth (2012) and sixth year (2014) were compared.

Maize cultivar (Xianyu 335) was manually sown, and covered with 2–3 cm soil layer. Spring drought is often a limiting factor for seed germination. Therefore, the crop was irrigated 55 mm water on plots using surface flood irrigation. After the pre-planting surface flooding, spring maize was generally dependent on rainfall water. All the plots received 225 kg N ha^-1^, 180 kg P_2_O_5_ ha^-1^, and 55 kg K_2_O ha^-1^. Fertilizer was broadcast evenly on the surface of plots before sowing, and then incorporated into the soil to 15 cm depth using rotary tillage. No additional fertilizer was then applied throughout the growing season. Plant row spacing was 50 cm, and plant-to-plant spacing was 30 cm, achieving a total plant density approximately of 66,700 plants ha^-1^. Subsoiling was done at 50 cm spacing using a tractor-driven subsoiler equipped with a chisel plow (1S-145, Zhongxin Agricultural Machinery Manufacturing Co., Ltd, Shandong, China). Subsoiling was conducted between two rows of plants at V6 stage in late May. To facilitate subsoiling, several items were repositioned to allow the proper functioning of subsoiler at differentdepths. Weeds were controlled using a herbicide (2,4-dichlorophenoxyacetic acid butylate) after sowing. The insecticide (phosphamidon) was sprayed on plants in middle June to avoid the yield losses due to insect attack.

### Measurement and calculation

#### Grain yield, biomass, and harvest index

Grain and biomass yield was determined by harvesting maize plants in middle rows on each plot at maturity. Plants were weighted separately after dividing them into grains and stalks. All the samples were oven-dried for 30 min at 105°C to quickly cease plant metabolic activities and then at 70°C to constant weight to attain the total aboveground biomass (kg ha^-1^). Thedry grain and stover weights were summed up to obtain the biomass yield. The air dried seed weight was adjusted to 14% moisture per plot and then converted to weights per hectare to calculate grain yield (kg ha^-1^). Harvest index (HI) was determined using the ratio of grain yield to total aboveground biomass yield.

#### Soil water storage, evapotranspiration, and water use efficiency

Soil water was determined gravimetrically by drying soil samples in an oven at 105°C for 12 h. The soil samples were taken fortnightly during the entire growing seasonfrom 0 to 120 cm soil layer, taken at 20 cm depth intervals. The volumetric water content (cm^3^ cm^-3^) was estimated by multiplying the soil water content (%) from each layer with soil bulk density. Soil water storage (SWS) was a product of volumetric water content by the thickness of soil layer.

In the study area, groundwater table was detected 7 m below the soil surface, aquifer recharge was then neglected. Surface runoff was reckoned to zero on the flat plots. The evapotranspiration (ET, mm) was determined using Eq 1 [23]:

$$ET\;=\;P\;+\;I\;+\;WS_{s}\;-\;WS_{h}$$ (1)

where *P* is precipitation (mm), *I* is irrigation quota (mm), measured using a flow meter at the recharging end of a hydrant pipe system, only 55 mm waterwas applied soon after sowing to guarantee germination. *WS*_*s*_ and *WS*_*h*_ are SWS (mm) in 0–120 cm depth before sowing and after harvesting, respectively.

Water use efficiency (WUE, kg ha^-1^ mm^-1^) was calculated as the ratio of grain yield (kg ha^-1^) to ET.

#### Field capacity, wilting point, and soil water deficit

Field capacity was used as an indicator for the upper limit of water that can be used by plants from soils. In this study, field capacitywas estimated before subsoilingby measuring the amount of water retained at an applied pressure of -33 kPa [24]. Wilting point (1500 kPa) was estimated before subsoiling from pressure plate analysis to be 50% of field capacity (30 kPa)[25]. Total available waterwas defined as the amount of soil water content between field capacity and wilting point. Measurements were taken from each layer at 20 cm increment to 120 cm depth. Using a management allowable depletion of 50% (which is commonly used for maize), the threshold *θ*_*t*_ was assumed to be halfway between the field capacity and wilting point. Therefore, it is assumed that 50% of total available water can be depleted from the root zone before soil water deficit(SWD) occurs [26]. SWD(mm) was calculated as:

$$SWD=\sum^{\;}Z_{i}{\left(\frac{\theta_{FC}+\theta_{WP}}{2}\;-\;\theta_{obs}\right)}_{i}$$ (2)

where *Z*_*i*_ is the thickness of soil layer *i* (cm), *θ*_*FC*_ and *θ*_*WP*_ are field capacity (cm^3^ cm^-3^) and wilting point (cm^3^ cm^-3^), respectively, *θ*_*obs*_ is the observed soil water content (cm^3^ cm^-3^) at a given layer. In this equation, SWD < 0 (desirable) means maize plantsarefree from water stress; SWD = 0 means plantsare in a balance between water supplyanduptake; SWD > 0 means plants have been suffering from water stress, and the severity intensifies with increasing SWD [27].

#### Yield associated with subsoiling depth

To determine the relationship between grain yield or biomass yield andsubsoiling depths, a modified power function was adopted using Eq 3:

$$y\;=\;a\;\times \;{\left(x\;+\;b\right)}^{c}$$ (3)

where *y* is grain yield or biomass yield (kg ha^-1^), *x* is subsoiling depth (cm), *a* and *b* are parameters to be fitted, *c* is an exponent to be estimated for controlling yield increase rate (kg ha^-1^ cm^-1^).

#### Evaluation index

An evaluation index was developed using yield- (grain yield, biomass yield, and HI), and water- (SWS, SWD, ET, and WUE) related factors as a whole. The index was used to determine the most effective tillage system for spring maize of northern China. Positive variables (i.e., grain yield, biomass yield, HI, SWS, and WUE) for assessments were standardized using Eq (4) while the negative variables (i.e., SWD and ET) were standardized using Eq (5) [28].

$$ax_{ij}=\frac{x_{ij}-x_{minj}}{x_{maxj}-x_{minj}}$$ (4)

or

$$ax_{ij}=\frac{x_{\text{max}j}-x_{ij}}{x_{maxj}-x_{minj}}$$ (5)

where *ax*_*ij*_ is a standardized value (0 ≤ *ax*_*ij*_ ≤1) for treatment *i* and variable *j*, *x*_*ij*_ is the corresponding actual value for treatment *i* and variable *j*, *x*_*max*_ and *x*_*min*_ are the maximum and minimum values for variables *j*.

After standardization, all the variables were presented in a range of 0 to 1. The significance and size of each variable used in assessmentdiffer in proportions. Therefore, weight coefficients were obtained using the method proposed by Qin(2013) [29] (shown in Eqs 6 and 7). Evaluation index is then determined as a linear combination of standardized value and weight coefficient for each variable using Eq (8).

$$\delta_{RMSEj}=\sqrt{\frac{1}{n}\overset{n}{\underset{i=1}{\sum }}{\left(ax_{ij}-\bar{ax_{ij}}\right)}^{2}}$$ (6)

where *δ*_*RMSEj*_ is the root mean squared error for variable *j*, *n* is the maximum number of treatments, *ax*_*ij*_ is the value obtained in Eqs 4 and 5.

$$Wt_{j}=\frac{\delta_{RMSE}}{\sum_{j=1}^{m}\delta_{RMSEj}}$$ (7)

where *Wt*_*j*_ is weight coefficient for variable *j*, *m* is the maximum number of variables, *δ*_*RMSEj*_ is the root mean squared error mentioned in Eq 6.

$$Index=\overset{m}{\underset{j=1}{\sum }}ax_{ij}\;\cdot \;Wt_{j}$$ (8)

### Statistical analyses

The data were subjected to univariatemodel of ANOVAin IBM SPSS Version 19.0 (IBM Corporation, Armonk, New York). All data were presented as mean ± SE(standard error). Treatment effects were determined using Fisher’s least significant difference (LSD) at *P*< 0.05. A modified power function was used to describe the relationship between yield andsubsoilingdepths. Allthe coefficients in the function were estimated by means of a non-linear regression based upon the Levenberg-Marquardt Algorithm. Due to significant treatment by year interactions for most of thevariables evaluated, the treatment effects were determined foreachsingle year.

## Results

### Soil water storage

**Fig 3:**
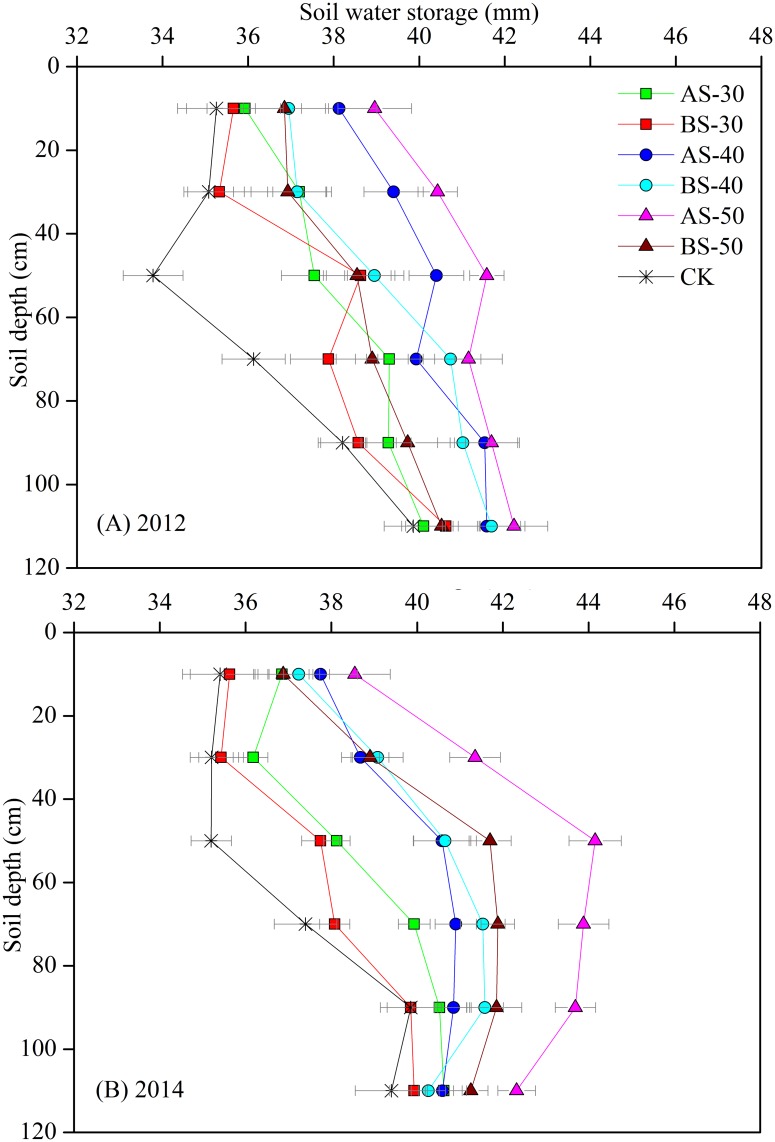
Spatial variations in soil water storage (SWS) in 0–120 cm soil layers at 20 cm interval. **(A)** SWSin2012 and **(B)** SWSin2014. Bars indicate standard errors (SE) at *P*< 0.05.

Subsoilingto40 and 50 cm depth with annual or biennial repetitions conserved more soil water (mm), especially in 20–80 cm soil layers, thanconventional tillage (CK) (Fig 3). In 2012, annual subsoiling to 50 cm (AS-50) increased SWS by an average of 10% in 0–20 cm soil depth (topsoil), 17% in 20–80 cm soil depth (medium soil layers), and 8% in 80–120 cm soil depth (deep soil layers), compared to the CK. Similarly, in 2014, AS-50 increased SWS by an average of 9% in topsoil, 20% in medium soil layers, and 9% in deep soil layers, compared to the CK. All treatments showed less effects on improving SWS in top and deep soils than the medium soil layers. In 2012, AS-40, BS-40, and BS-50 increased SWS in medium soil layers by an average (mm) of 14%, 11%, and 9%, respectively, compared to the CK. Similarly, in 2014, AS-40, BS-40, and BS-50 increased SWS in medium soil layers by an average of 11%, 13%, and 14%, respectively, compared to the CK. AS-30 and BS-30 had very limited SWS improvement effect across the 0–120 cm soil layers compared to the CK.

### Soil water deficit

**Fig 4:**
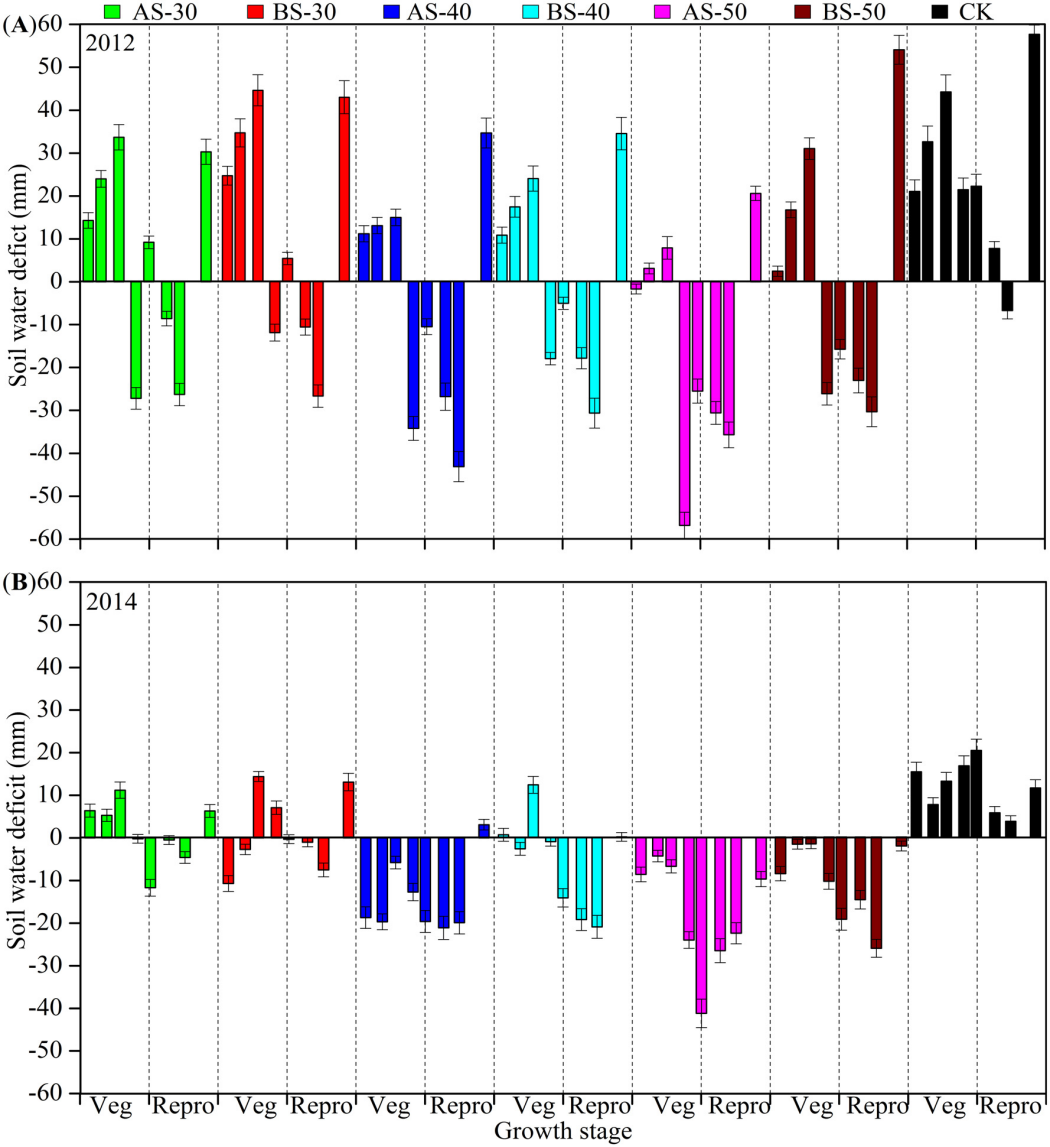
Soil water deficit (SWD) for each treatment, separated by growth stages. **(A)** SWD in 2012, and **(B)** SWD in2014. The horizontal lines across the Fig indicate soil water deficit = 0, while the bars indicate standard errors at *P*< 0.05 (SE).

In this study, soil water deficit (SWD, mm) was > 0 (undesirable) from sowing (late April) to jointing (middle June) in 2012 season for most of the treatments, indicatingthatspring drought extendedtojointingstage(Fig 4). While in the same period of 2014, AS-40, AS-50 and BS-50 effectively prevented maize from spring drought due to negativeSWD (desirable). In late jointing (late June) to filling stage (late August), water stresswas significantly alleviated indicated by the lowernegative SWD, except CK, BS-30 and AS-30 treatments.

In the critical growth period (mid July), when plant entered into reproductive phase from vegetative phase, SWD of AS-50 varied from -25.5 to -41.3 mm (the lowest values among the treatments) in 2012 and 2014, respectively, followed by BS-50and AS-40with SWD varying from -15.8 to -19.6mmin 2012, and -10.6 to -19.3 mmin2014, respectively. In contrast, BS-40 and BS-50 showed SWD slightly > 0 (1.1to 1.9 mm) in 2012, however, the SWD was reduced to -10.6 to -5.7 mm in 2014. The significantly > 0 values of SWDobservedin both the years for AS-30, BS-30, and CK indicated a persistent water stress for spring maize with shallow subsoiling and the conventional tillage.

### Yield, evapotranspiration, and water use efficiency

Annual subsoilinghas shown the yield advantage over biennial subsoiling, whereas deep subsoiling over-yielded the shallower one on the sandy loam in this study (Table 2). Grain yields with annual subsoiling were 11,731 and 11,814 kg ha^-1^ during 2012 and 2014, respectively, or increased by 8% and 6% as compared to biennial subsoiling. On the basis of subsoiling depth, deep subsoiling(50 cm) produced yields of 12,014 and 12,296 kg ha^-1^ during 2012 and 2014, respectively, or increased by 16% and 18% as compared to shallow subsoiling (30 cm). The maximum grain yield was produced by AS-50. Similar trends were also observed in the case of biomass yield (aboveground). On average, AS-50 produced the maximum biomass yield, followed by BS-50. In the present study, the significantly lower HI was observed in the case of AS-50as compared to BS-50 and AS-40. Deep subsoilingincreasedET due to an increase of water availability. In the present study, deep subsoiling increased ET by 13% (2012) and 9% (2014), compared to shallow subsoiling. Similarly, annual subsoilingincreasedET by 7% compared to biennial subsoiling. The present study also compared WUE under different tillagesystems. Although WUE differednon-significantly during 2012, it showed significant effects in 2014. This significance of WUE was attributed to the two years of subsoiling. WUE with deep subsoiling showed an increase by 8% as compared to shallow subsoiling, while medium subsoiling did not differ from shallow subsoiling in both the years.

**Table 2:** Grain yield, biomass yield, evapotranspiration (ET), water use efficiency (WUE), and harvest index (HI) as affected by subsoiling depth and frequency, at Jiaozuo experiment station during 2012 and 2014.

Year	Treatment	Yield	Biomass	ET	WUE	HI
		– – – kg ha^-1^ – – –		– mm –	kg ha^-1^ mm^-1^	
**2012**	AS-30 ^a^	10831 c ^b^	23248 c	314.6 de	34.4 a	0.47 b
	BS-30	9874 d	22757 c	303.2 e	32.6 b	0.43 c
	AS-40	12083 ab	24288 b	351.2 ab	34.4 a	0.50 a
	BS-40	11123 c	23333 c	324.9 cd	34.2 a	0.48 ab
	AS-50	12278 a	28568 a	361.2 a	34.0 a	0.43 c
	BS-50	11749 b	24887 b	337.3 bc	34.8 a	0.47 b
	CK	9510 d	20634 d	303.2 e	31.4 c	0.46 b
**2014**	AS-30	10699 d	23158 d	354.8 b	30.2 c	0.46 cd
	BS-30	10058 e	22837 d	329.4 c	30.5 c	0.44 d
	AS-40	12132 ab	23480 cd	386.9 a	31.4 bc	0.52 a
	BS-40	11314 c	24072 bc	373.6 ab	30.3 c	0.47 bc
	AS-50	12611 a	26151 a	389.3 a	32.4 ab	0.48 bc
	BS-50	11981 b	24305 b	358.5 b	33.4 a	0.49 b
	CK	9515 f	21445 e	330.1 c	28.8 d	0.44 d

^a^ AS stands for annual subsoiling, and BS, biennial subsoiling, and 30, 40, 50 are the subsoiling depths (cm); CK, the conventional tillage.

^b^ Different letters in each section of a column declare significant differences at *P*< 0.05.

### Relationshipsbetween yield and subsoiling depth

**Fig 5:**
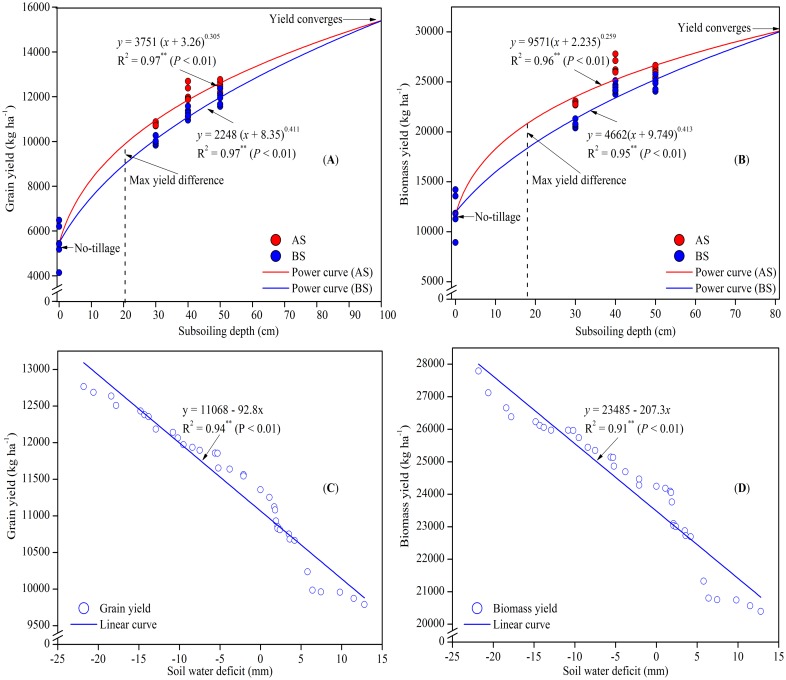
Yield response of spring maize to subsoiling depth and soil water deficit. **(A)** Relationship between grain yield and subsoiling and **(B)** between biomass yield and subsoiling depth was fitted to a modified power function (*P*< 0.01);**(C)** relationship between grain yield and soil water deficit (SWD) and **(D)** between biomass yield and SWD was fitted to a fitted to a negative linear correlation (*P*< 0.01). AS: annual subsoiling; BS: biennial subsoiling.

A modified power function was fitted to describe the relationship between yieldandsubsoiling depth (*P*< 0.01) (Fig 5). The exponent, *c*, was < 1 for both annual and biennial treatments, exhibiting a concave curve. The larger values of *c* with biennial subsoilingcompared to annual subsoiling indicated a higher rate of yield increase with biennial subsoiling. The similar exponent, *c*, both in the case of biomass yield and grain yield indicated a similar rate of yield increase withsubsoiling depths. The modified power function observed the maximum grain yield difference (906 kg ha^-1^) and biomass yield difference (2470 kg ha^-1^) between annual and biennial subsoiling at 20 cm and 17 cm subsoiling depths, respectively. The difference then gradually reduced to a convergence point at 100 cm (for grain yield) and 83 cm (for biomass yield) subsoiling depths, respectively.

## Discussion

In this study, we found that subsoiling treatments merely hadsignificant effects on SWS both in topsoil (0–20 cm), and in deep soil (80–120 cm). However, obvious differences were observed in soil layers of 20–80 cm. Previous study on rainfed durum wheat reported that subsoiling to 60 cm produced higher SWS in 40–60 cm on a silty clay soil while a non-significant effect was observed in deeper layers [14]. Another study on semi-arid winter sorghum on a silty loam soil showed that deep subsoiling conserved higher SWS in top 60 cm soil layer as compared to medium and shallow subsoiling [30]. The higher SWS values achieved bydeepsubsoiling provided more total available water for uptake than medium and shallowsubsoiling.

According to Dejonge et al. (2015) [25], mid-value between wilting point and field capacity can be used as a threshold to determine whether current soil water status is desirable or undesirable to maize. Soil water deficit (SWD) < 0 (desirable) indicated SWS was currently adequate for plant use while SWD > 0 (undesirable) meant SWS was deficitfor uptake. SWD = 0 meant a balance between water supply and uptake [27]. The findings of SWD (an indicator used to estimate the severity of water stress) confirmed ourhypothesis. In May of 2012, most of the treatments had SWD > 0 on a sandy loam due to spring drought. Similar effects of spring drought on a light loam soil were reported on winter wheatin North China Plain [31]. During reproductive phase(July), AS-50achieved the lowest SWD among treatments. Thelower SWD with AS-50 effectively prevented plants from water stress in mid-season [21]. The lower SWD and higher yield achieved by AS-50 against the other tillage treatments validated our hypothesis that higher yield was closely associated with lower SWD. However, the SWD values ≈ 0 in the case of AS-30 and BS-30indicated that the AS-30 and BS-30 made the plant more vulnerable to water deficit as compared to deep subsoiling.

Previous studies under non-irrigated condition showed thatsubsoiling improved grain yield by up to 46% as compared to conventional tillage. However, the differences between subsoiling and conventional tillage became non-significant under irrigated conditions [20]. Generally, on a sandy loamsoil, theconventional tillage resulted in a yield decrease when water stress was a limiting factor [32]. The present study was conducted innon-irrigated conditions on a sandy loam soil, and rainwater was a major water source for plants. During dry season, annual deep subsoiling with no irrigation on a silt loam made a good use of conserved rainwater and consequently produced high yield [33]. Several previous studies found that differences in grain yield between annual and biennial subsoilingwere non-significant [34], yet, in the case of rainfed cultivation, yield with annual subsoilingwas slightly higher than that of biennial subsoiling [35]. In this study, annual subsoilingshowed significant yield advantage over biennial subsoiling in both the years under rainfed condition. Busscher et al. (1988) [36] reported that annual subsoiling offered little benefits in first several years and thedifferences in grain yield would be diminished if sufficient water is available to fulfill the crop’s water requirement [37]. Our result showed that AS-50 and AS-40can significantly improve grain yield, especially during uneven rainfall season. This improvement can be attributed to the capacity of AS-50 to utilize soilwater stored in deep layers during short-term water stressepisode [14].

In this study, both grain and biomass yields were proportional to subsoiling depths, butyield increase rate (kg ha^-1^ cm^-1^) gradually decreased with depths. The decrease in yield increase rate with the increasingsubsoiling depths was probably determined by crop biophysical limits [38]. In addition, the present study confirmed a negative linear correlation between yield and SWD(*P*< 0.01) (Fig 5). Per mm reduction of SWD resulted in 93 kg ha^-1^grain yield and 207 kg ha^-1^ biomass yield increment, respectively. The results were in consensus with the observations reported by Heatherly and Spurlock, (2001)[39] on a loamy clay soil.

Also, we have quantified several important water- and yield-related factors to ensure the comparison of effects in a quantitative manner. The indices are in the range of 0 to 1, the higher the value gets, the more effective the treatment is(Table 3). The maximum evaluation index (0.69 out of 1.0) in the case of annualsubsoiling to 50 cm depth demonstrated that AS-50 is the most effective treatmentfor increasing productivity and mitigating the effects of water stress in spring maize of northern China.

**Table 3:** Comprehensive assessment for different tillage treatments using water- and yield-related data standardized in a range of 0 to 1 to compare treatment effects quantitatively.

Treatment ^a^	SWS ^b^	GY	BY	WUE	HI	SWD	ET	Index ^c^
**AS-30**	0.42	0.43	0.34	0.57	0.31	0.42	0.68	0.45 c ^d^
**BS-30**	0.28	0.15	0.28	0.39	0	0.32	0.99	0.34 d
**AS-40**	0.78	0.88	0.45	0.73	0.88	0.8	0.1	0.66 b
**CS-40**	0.59	0.58	0.42	0.56	0.44	0.59	0.44	0.51 c
**AS-50**	1	1	1	0.8	0.19	1	0.08	0.69 a
**CS-50**	0.67	0.8	0.56	1	0.5	0.68	0.46	0.65 b
**CK**	0	0	0	0.03	0.13	0	0.99	0.18 e
**Weight coefficient**	0.135	0.148	0.149	0.117	0.179	0.118	0.156	

^a^ Treatments include AS-30, annual subsoiling to 30 cm depth, BS-30, biennial subsoiling to 30 cm depth, AS-40, annual subsoiling to 40 cm depth, BS-40, biennial subsoiling to 40 cm depth, AS-50, annual subsoiling to 50 cm depth, BS-50, biennial subsoiling to 50 cm depth, CK, the conventional tillage.

^b^ Variables include SWS, soil water storage, GY, Grain yield, BY, biomass yield, WUE, water use efficiency, HI, harvest index, SWD, soil water deficit, ET, evapotranspiration.

^c^ Index is created using the variables as functional components, tillage treatment with higher value is considered more effective.

^d^ Means with different letters in the same column indicate significant difference at *P*< 0.05.

## Conclusion

Annual spring inter-row subsoiling to 50 cm depth (AS-50) significantly improvedthebiomass and grain yields by increasing SWS in 20–80 cm soil depth and decreasing SWD in the critical growth period of spring maize.Grainand biomass yield response to subsoiling depths can be quantified as a power function where the yield is positively proportional to subsoiling depths, whereas the relationship between yield and soil water deficit is fitted to a linear function where the yield is negatively proportional to soil water deficit. The improved SWS and reduced SWD by AS-50 allowed spring maize in a well soil water statusespeciallyinthe early stage of crop growth when spring drought frequently occurred. Furthermore, short-term water stressepisode could be induced during the middle stage of crop growth by the conventional tillage or shallow subsoiling systems, whereasdeepsubsoiling in combination with annual repetition was shown to alleviateit. The highest evaluation index of the annual spring deep inter-row subsoiling clearly demonstrates that AS-50 can play an important role in the development of improved water conservation practicesallowing to reduce SWD, increase SWS, and boostyield of spring maize on a sandy loam in northern China.
